# Perceptions of the Transition Process From Senior High School to First-Year Medical School at Chiang Mai University

**DOI:** 10.7759/cureus.96354

**Published:** 2025-11-08

**Authors:** Theerapat Inta, Wiyada Dankai, Manoch Chokjamsai, Komson Wannasai

**Affiliations:** 1 Department of Pathology, Faculty of Medicine, Chiang Mai University, Chiang Mai, THA; 2 Department of Forensic Medicine, Faculty of Medicine, Chiang Mai University, Chiang Mai, THA

**Keywords:** first-year medical students, learner transition, medical education, psychosocial stress, questionnaire

## Abstract

Introduction and aim

The transition from high school to university can present various challenges for students, including adapting to new educational systems, cultural settings, and financial responsibilities. These changes often lead to feelings of anxiety and stress, potentially hindering students’ success. To facilitate a smoother transition, educational institutions should offer support services such as counseling, guidance, and financial aid. This study aimed to explore the transition experiences of first-year medical students at Chiang Mai University, Faculty of Medicine, in the 2023 academic year.

Materials and methods

A questionnaire using a five-point Likert scale was employed to gather insights into students’ concerns during this pivotal period, with the research conducted within the first month of the academic year, focusing on aspects such as environmental adjustments, financial pressures, social interactions, and academic challenges.

Results

Students are most anxious about upcoming examinations, with a rating of 3.66, closely followed by concerns regarding lecture content at 3.49. Regional differences reveal that students from outside Northern Thailand exhibit heightened levels of concern in various areas, including social integration, adapting to new environments, forming new friendships, understanding lectures, preparing for exams, and academic performance.

Discussion

The transition from high school to medical school can be a challenging and emotionally taxing experience. Academic achievement is critically important in how students perceive the varied obstacles they face. Hence, it is of utmost importance to develop interventions that specifically target the enhancement of academic performance and psychological well-being to ensure their success.

Conclusion

It is clear that students, particularly those from outside Northern Thailand, experience heightened anxiety about exams and challenges in adapting to new academic and social environments. These findings demonstrate the need for targeted interventions that address both academic stress and psychological well-being. Specifically, support systems focusing on exam preparation, social integration, and mental health are essential to help students navigate the transition more smoothly. By implementing these targeted strategies, we can improve the overall transition experience from high school to medical school, especially for students facing regional and environmental challenges.

## Introduction

Transition is the internal process in the mind that occurs when students undergo change and transition from the familiar to the unknown, responding to environmental, financial, social, and academic challenges [1,2]. For instance, during the transition phase from senior high school to first-year medical school, students will encounter a new educational system, an unfamiliar culture (such as forming new relationships with peers, university support, and academic staff, as well as flatmates), presentations and examinations, expectations, and pressure from multiple sources. Many students also face a financial burden, requiring them to work part-time and take on loans to support their university education [3]. Such changes cause stress, anxiety, homesickness, fear of being ignored, feelings of embarrassment, confusion, and lack of self-confidence, potentially leading to depression [4]. Thus, both schools and universities should work together to support students in systematically adapting to the new environment to achieve a smooth and successful transition [5].

In a 2016 research study, Juma et al. reported the establishment of the Manchester Access Programme, which is delivered by the University of Manchester [5]. The aim of the scheme was to identify underprivileged students who may be at increased risk of dropping out and encourage them to prepare for university by receiving counselling and advice on university life, addressing the financial burden annually with a support system involving teachers and seniors. Students would then be better able to cope with the transition phase smoothly and successfully, ultimately reducing the likelihood of students dropping out of university.

In a recent study by Malau-Aduli et al. on the perceptions and processes influencing the transition of medical students from preclinical to clinical training, questionnaire responses were obtained from 141 students [6]. The findings revealed that the majority of students were worried about the workload and knowledge gaps, such as the application of basic knowledge in health sciences and theoretical knowledge in practice with real patients. Likewise, during the interviews and a group discussion with 12 individuals, it was found that workload, socialization, and knowledge gaps were regarded as the major issues for students. Therefore, educators should systematically provide support from peers, seniors, graduates, tutors, and teachers to minimize such problems and encourage students to develop their learning process and adapt to the content little by little, allowing them to effectively apply basic to advanced knowledge.

Chiang Mai University’s Faculty of Medicine admits students through the Thailand Central Admission System (TCAS), comprising the following three rounds: the portfolio round, which evaluates applicants based on high school achievements, standardized tests, and interviews; the quota round, aimed at increasing rural healthcare providers by prioritizing students from the Northern Development Region with specific exam scores; and the direct admission round, which selects students nationwide based solely on A-level scores. Upon admission, students can choose from various housing options and enroll in core courses, such as biochemistry, genetics, and developmental biology, in addition to elective subjects. They can also apply for scholarships based on need. During their initial month, students focus on foundational academic subjects and participate in extracurricular activities organized by Medical Student Affairs, fostering community, personal growth, and networking.

Medical students at Chiang Mai University often encounter a range of challenges during their transition into medical training, including environmental, financial, social, and academic difficulties. To achieve a comprehensive understanding of this transition, the research team conducted a structured questionnaire survey to test the hypothesis that students experience significant concerns across these four domains during their first month of medical school. The survey assessed students’ perceptions and levels of concern within each domain during the initial phase of the 2023 academic year. This study is particularly significant because it specifically focuses on the experiences of first-year medical students transitioning directly from high school and compares the concerns of students from regional areas with those from urban or other backgrounds. As such, this research aimed to fill an important gap in the existing literature by offering comprehensive details about the unique transitional experiences of medical students at Chiang Mai University.

## Materials and methods

Sampling and participants

This study was conducted in the 2023 academic year on a sample of 250 first-year medical students enrolled in the medical program at the Faculty of Medicine, Chiang Mai University. These medical students have studied for one month before completing the questionnaire. The research sample included first-year medical students who were 12th-grade high school students in the previous academic year (2022) and had been medical students for one month after the start of the first semester. These students expressed a willingness to participate in a self-assessment questionnaire. According to a study by Louangrath, the minimum sample size for survey studies, particularly for a five-point Likert scale, is 30 [7].

The Research Ethics Committee of the Faculty of Medicine, Chiang Mai University, reviewed the protocol and granted an exemption for this cross-sectional study (#FAC-MED-2566-0356).

Data collection and analysis

The team took certain measures to minimize potential conflicts of interest between the research team and the participants. These measures included providing participants with a comprehensive overview of the study and actively recruiting volunteers for the research project. Additionally, the team provided a Google Forms (Mountain View, CA: Google LLC) questionnaire in the Thai language for students who expressed interest in participating. The team clarified the study subject, the data collection method, and the advantages and disadvantages of participating in the research project. To demonstrate their willingness to respond to the questionnaire, it was necessary for the participants to meet the following two requirements: completing the Google Form to express their agreement to participate and subsequently submitting it using Google Forms. Participants who declined to finish the questionnaire would be ineligible to proceed. The questionnaire was administered in an anonymous manner, with only the participants’ gender being disclosed. The sampling method used to invite participants for the questionnaire involved generating a QR code that provided access to the survey. After the class was over, the QR code was displayed in front of the students. They were invited to voluntarily complete the questionnaire at their convenience. This approach ensured that participation was entirely voluntary while allowing for a broad distribution of the questionnaire among the class (table in appendix).

To ensure the validity of the research, a questionnaire was developed to gather students' perspectives on the transitional process. Our team specifically developed the questionnaire for this research. We employed a five-point Likert scale exclusively; no third-party or licensed instruments were utilized, and consequently, no licensing was required [8]. This questionnaire was created based on a thorough examination of the existing literature, input from two students who reviewed the questionnaire, feedback from faculty members from two different departments who conducted a peer review, and insights provided by an expert on medical education [1,2,5,6]. We begin by thoroughly reviewing relevant studies, articles, and reports to identify key themes, concepts, and variables related to our research topic. From this review, we extracted important questions, measurement scales, and findings that had been previously used, and then selected and adapted the most relevant and reliable items to suit our specific research goals. Finally, we organize these questions in a logical manner to ensure clarity, consistency, and ease of understanding. A pretest of the questionnaire was conducted with 20 medical students who were not part of the study group. This pre-testing helped to assess the clarity, relevance, and overall functionality of the questionnaire before it was administered to the main study participants. The participants rated their experiences using a 20-item questionnaire based on a five-point Likert scale, ranging from "strongly unconcerned" to "strongly concerned" [8]. The results of the mean score for each question were subsequently documented. The survey required students to express their thoughts on concerns about the transition from high school to medical school. Using a five-point Likert scale (1=strongly unsatisfied, 2=unsatisfied, 3=neither satisfied nor unsatisfied, 4=satisfied, and 5=strongly satisfied), students were asked about the following four main aspects: environment, financial, social, and academic.

The data were subjected to analysis using STATA version 16 (College Station, TX: STATA Corp.), with mean, standard deviation (SD), median, and the interquartile range (IQR) serving as the statistical measures. The Mann-Whitney U (Wilcoxon rank-sum) test was used to evaluate the significance of differences in median values between students from Northern Thailand and those from other regions within the country, as well as between male and female students. The threshold for statistical significance was deemed acceptable at a significance level of p<0.05. Cronbach’s alpha was used to assess the reliability of the questionnaire.

## Results

Data collection and analysis

The response rate for the survey was 60.9% (n=134), with a mean age of 18.36±0.66 (17-21) years. The male and female students were 79 (58.96%) and 55 (41.04%), respectively. Ninety-one students (67.91%) came from the northern part of Thailand, and 43 (32.09%) from other regions. To maintain anonymity, regional data were collected in a way that did not include identifying information and were aggregated during analysis. Detailed information on the participants’ demographics is presented in Table 1.

**Table 1:** Demographics of participants (n=134).

Parameters	n=134	Percentage
Age (years)		
17	3	2.24
18	89	66.42
19	35	26.12
20	5	3.73
21	2	1.49
Gender		
Male	79	58.96
Female	55	41.04
Region		
Northern Thailand	91	67.91
Non-Northern Thailand	43	32.09

Survey results

The mean and median scores for all Likert-scale statements are summarized in Table 2 [8]. The median scores for the four assessed domains were 3 for environment, 2 for financial, 3 for social, and 3 for academic. Overall, in all the domains, academic concerns were the most prevalent, with an average rating of 3.14. Environment ranked second highest, with a score of 2.87. Statistically, female medical students were more concerned than their male counterparts (mean: 3.08 vs. 2.71; median: 3 vs. 3, p=0.0001). When comparing students from Northern Thailand with those from other parts of the country, it was observed that students from this region tended to be less concerned about almost every aspect than those who came from other parts of the country (mean: 3.08 vs. 2.76; median: 3 vs. 3, p=0.0001). The Cronbach's alpha coefficient of the overall questionnaire was 0.935.

**Table 2:** Mean and median scores for the Likert-scale statements by domain. Q1=first quartile; Q3=third quartile

Questions		Mean (SD)	Median (Q1, Q3)	IQR
Domain 1: environment		2.87 (1.25)	3 (2, 4)	2
Q1	I feel homesick.	2.94 (1.16)	3 (2, 4)	1
Q2	I am afraid of being separated from my family.	2.43 (1.10)	2 (2, 3)	1
Q3	I am worried about being overlooked by people around me.	3.28 (1.26)	3 (2, 4)	2
Q4	I am afraid that I will not be able to adapt to the university environment.	2.83 (1.32)	3 (2, 4)	2
Domain 2: financial		2.31 (1.23)	2 (1, 3)	2
Q5	I am worried about paying the tuition fees.	1.79 (1.01)	1 (1, 2)	1
Q6	I worry about my daily expenses.	2.82 (1.22)	3 (2, 4)	2
Domain 3: social		2.58 (1.25)	3 (1, 3)	2
Q7	I am worried about making new friends.	3.08 (1.21)	3 (2, 4)	2
Q8	I am worried about approaching or consulting with a teacher at the university.	3.01 (1.23)	3 (2, 4)	2
Q9	I am worried about approaching or consulting university officials.	2.67 (1.21)	3 (2, 3)	1
Q10	I am worried about living with dorm mates.	2.00 (1.17)	2 (1, 3)	2
Q11	I feel lonely and alone.	2.14 (1.06)	2 (1, 3)	2
Domain 4: academic		3.14 (1.29)	3 (2, 4)	4
Q12	I am worried about whether I will be able to be a part of the teaching or not.	2.65 (1.20)	3 (2, 3)	1
Q13	I am worried about adjusting to the teaching environment.	2.74 (1.24)	3 (2, 4)	1
Q14	I am worried I will not be able to understand the content well enough.	3.49 (1.19)	4 (3, 4)	1
Q15	I feel like I must bear the expectations of others when it comes to my studies.	2.74 (1.29)	3 (2, 4)	2
Q16	I am worried about my ability to study.	3.23 (1.30)	3 (2, 4)	2
Q17	I am afraid the content will be too difficult for me.	3.19 (1.27)	3 (2, 4)	2
Q18	I am worried about the upcoming exam.	3.66 (1.16)	4 (3, 5)	2
Q19	I am worried about giving a speech or talk for the biochemistry group project to the audience, including answering the teacher’s questions in front of the class.	3.14 (1.23)	3 (2, 4)	2
Q20	I am worried about the results not being good enough, which is just as I feared at the outset.	3.43 (1.35)	4 (2, 5)	3

Environment

As can be observed from Table 2, large numbers of students were worried about being overlooked by people around them, with a median score of 3 and an average score of 3.28. Not only were many afraid of being separated from their family, with an average rating of 2.43 points (median 2), but they also needed time to adjust to the new environment and homesickness, with average scores of 2.94 (median 3) and 2.83 (median 3), respectively. The Cronbach's alpha coefficient of the questionnaire in this part was 0.655. As illustrated in Table 3, significant gender differences were observed in the environment domain related to anxiety about being separated from family, with more females reporting that they felt nervous (p=0.0024) and feared being ignored by their community (p=0.0133) [8]. Similarly, females expressed greater anxiety than their male counterparts regarding the transition from high school to university (p=0.0316). In addition, as displayed in Table 4, students from different regions of Thailand showed distinct variations in their experiences within a new environment [8]. Individuals from areas outside Northern Thailand reported greater anxiety about social exclusion (p=0.0033) and had more difficulty adjusting to their new surroundings (p=0.0166). These findings were statistically significant.

**Table 3:** Median and mean scores for the Likert-scale statements in relation to gender. Mann-Whitney U test was used; test statistics are shown as standardized Z-values from STATA output (College Station, TX: STATA Corp.). Q1=first quartile; Q3=third quartile

Questions		Male			Female		Mann-Whitney U	p-Value
		Median (Q1, Q3; IQR)	Mean (SD)	Median (Q1, Q3; IQR)		Mean (SD)	Z-value	
Domain 1: environment								
Q1	I feel homesick.	3 (2, 4; 2)	2.80 (1.13)	3 (3, 4; 1)		3.16 (1.18)	2.023	0.0431
Q2	I am afraid of being separated from my family.	2 (2, 3; 1)	2.20 (1.08)	3 (2, 4; 2)		2.76 (1.07)	3.033	0.0024
Q3	I am worried about being overlooked by people around me.	3 (2, 4; 2)	3.04 (1.31)	4 (3, 5; 2)		3.62 (1.13)	2.303	0.0133
Q4	I am afraid that I will not be able to adapt to the university environment.	2 (1, 4; 3)	2.63 (1.39)	3 (2, 4; 2)		3.11 (1.16)	2.149	0.0316
Domain 2: financial								
Q5	I am worried about paying the tuition fees.	2 (1, 3; 2)	1.91 (1.03)	1 (1, 2; 1)		1.62 (0.97)	-1.977	0.0480
Q6	I worry about my daily expenses.	3 (2, 4; 2)	2.73 (1.20)	3 (2, 4; 2)		2.95 (1.27)	1.021	0.3073
Domain 3: social								
Q7	I am worried about making new friends.	3 (2, 4; 2)	2.85 (1.28)	3 (3, 4; 1)		3.42 (1.03)	2.643	0.0082
Q8	I am worried about approaching or consulting with a teacher at the university.	3 (2, 4; 2)	2.86 (1.22)	3 (2, 4; 2)		3.24 (1.22)	1.807	0.0708
Q9	I am worried about approaching or consulting university officials.	3 (2, 3; 1)	2.59 (1.17)	3 (2, 4; 2)		2.78 (1.27)	0.766	0.4437
Q10	I am worried about living with dorm mates.	2 (1, 3; 2)	2.11 (1.19)	1 (1, 3; 2)		1.84 (1.13)	-1.609	0.1075
Q11	I feel lonely and alone.	2 (1, 3; 2)	2.19 (1.14)	2 (1, 3; 2)		2.07 (0.94)	-0.261	0.7943
Domain 4: academic								
Q12	I am worried about whether I will be able to be a part of the teaching or not.	2 (2, 3; 1)	2.49 (1.16)	3 (2, 4; 2)		2.87 (1.20)	1.848	0.0647
Q13	I am worried about adjusting to the teaching environment.	3 (1, 3; 2)	2.58 (1.25)	3 (2, 4; 2)		2.96 (1.21)	1.787	0.0740
Q14	I am worried I will not be able to understand the content well enough.	3 (2, 4; 2)	3.23 (1.20)	4 (3, 5; 2)		3.85 (1.08)	3.051	0.0023
Q15	I feel like I must bear the expectations of others when it comes to my studies.	2 (2, 3; 1)	2.49 (1.20)	3 (2, 4; 2)		3.09 (1.35)	2.548	0.0108
Q16	I am worried about my ability to study.	3 (2, 4; 2)	2.99 (1.24)	4 (3, 5; 2)		3.58 (1.32)	2.707	0.0068
Q17	I am afraid the content will be too difficult for me.	3 (2, 4; 2)	2.96 (1.21)	4 (3, 5; 2)		3.51 (1.29)	2.539	0.0111
Q18	I am worried about the upcoming exam.	3 (3, 4; 1)	3.45 (1.20)	4 (3, 5; 2)		3.96 (1.04)	2.437	0.0148
Q19	I am worried about giving a speech or talk for the biochemistry group project to the audience, including answering the teacher’s questions in front of the class.	3 (2, 4; 2)	2.92 (1.20)	4 (3, 4; 1)		3.45 (1.21)	2.572	0.0101
Q20	I am worried about the results not being good enough, which is just as I feared at the outset.	3 (2, 4; 2)	3.25 (1.33)	4 (3, 5; 2)		3.69 (1.35)	2.009	0.0445

**Table 4:** Median and mean scores for the Likert-scale statements in relation to region. Mann-Whitney U test was used; test statistics are shown as standardized Z-values from STATA output (College Station, TX: STATA Corp.). Q1=first quartile; Q3=third quartile

Questions		Non-northern		Northern		Mann-Whitney U	p-Value
Domain 1: environment		Median (Q1, Q3; IQR)	Mean (SD)	Median (Q1, Q3; IQR)	Mean (SD)	Z-value	
Q1	I feel homesick.	3 (3, 4; 1)	3.14 (0.86)	3 (2, 4; 2)	2.86 (1.27)	-1.270	0.2040
Q2	I am afraid of being separated from my family.	3 (2, 3; 1)	2.51 (1.12)	2 (2, 3; 1)	2.40 (1.10)	-0.645	0.5192
Q3	I am worried about being overlooked by people around me.	4 (3, 5; 2)	3.74 (1.16)	3 (2, 4; 2)	3.05 (1.26)	-2.725	0.0033
Q4	I am afraid that I will not be able to adapt to the university environment.	3 (2, 4; 2)	3.21 (1.17)	2 (1, 4; 3)	2.65 (1.35)	-2.396	0.0166
Domain 2: financial							
Q5	I am worried about paying the tuition fees.	1 (1, 2; 1)	1.67 (0.87)	1 (1, 3; 2)	1.85 (1.07)	0.607	0.5440
Q6	I worry about my daily expenses.	3 (2, 4; 2)	2.91 (1.09)	3 (2, 4; 2)	2.78 (1.29)	-0.625	0.5321
Domain 3: social							
Q7	I am worried about making new friends.	4 (3, 4; 1)	3.51 (1.01)	3 (2, 4; 2)	2.88 (1.25)	-2.815	0.0049
Q8	I am worried about approaching or consulting with a teacher at the university.	3 (2, 4; 2)	3.26 (1.27)	3 (2, 4; 2)	2.90 (1.19)	-1.524	0.1274
Q9	I am worried about approaching or consulting university officials.	3 (2, 4; 2)	2.88 (1.29)	3 (2, 3; 1)	2.57 (1.17)	-1.244	0.2136
Q10	I am worried about living with dorm mates.	2 (1, 3; 2)	1.95 (1.02)	2 (1, 3; 2)	2.02 (1.24)	-0.084	0.9332
Q11	I feel lonely and alone.	2 (2, 3; 1)	2.33 (1.06)	2 (1, 3; 2)	2.05 (1.06)	-1.478	0.1393
Domain 4: academic							
Q12	I am worried about whether I will be able to be a part of the teaching or not.	3 (2, 4; 2)	2.86 (1.23)	3 (2, 3; 1)	2.55 (1.17)	-1.375	0.1691
Q13	I am worried about adjusting to the teaching environment.	3 (2, 4; 2)	2.95 (1.25)	3 (2, 3; 1)	2.64 (1.23)	-1.327	0.1845
Q14	I am worried I will not be able to understand the content well enough.	4 (3, 5; 2)	3.77 (1.09)	3 (3, 4; 1)	3.35 (1.21)	-1.836	0.0664
Q15	I feel like I must bear the expectations of others when it comes to my studies.	3 (2, 4; 2)	2.74 (1.29)	3 (2, 4; 2)	2.74 (1.30)	-0.012	0.9909
Q16	I am worried about my ability to study.	4 (3, 4; 1)	3.51 (1.18)	3 (2, 4; 2)	3.10 (1.33)	-1.715	0.0863
Q17	I am afraid the content will be too difficult for me.	4 (3, 4; 1)	3.47 (1.18)	3 (2, 4; 2)	3.05 (1.29)	-1.711	0.0872
Q18	I am worried about the upcoming exam.	4 (3, 5; 2)	4.02 (0.99)	4 (3, 5; 2)	3.49 (1.21)	-2.380	0.0173
Q19	I am worried about giving a speech or talk for the biochemistry group project to an audience, including answering the teacher’s questions in front of the class.	3 (3, 5; 2)	3.42 (1.20)	3 (2, 4; 2)	3.01 (1.22)	-1.632	0.1028
Q20	I am worried about the results not being good enough, which is just as I feared at the outset.	4 (3, 5; 2)	3.84 (1.33)	3 (2, 4; 2)	3.24 (1.33)	-2.607	0.0091

Financial

As indicated in Table 2, while a few participants expressed anxiety regarding tuition fees, with an average rating of 1.79 (median 1), the majority of students exhibited greater apprehension about their daily expenses, with an average score of 2.82 (median 3). This indicates that most students were somewhat concerned or worried about managing their daily financial needs, reflecting a common financial stressor among the participants. The Cronbach's alpha coefficient for the questionnaire in this part was 0.576.

Social

According to Table 2, the social behaviors eliciting the highest levels of concern were initiating new friendships and seeking guidance from a teacher, with average scores of 3.08 (median 3) and 3.01 (median 3), respectively. Following closely behind was the act of approaching university officials, with an average rating of 2.67 (median 3). Conversely, social isolation and interactions with dorm mates were indicated to be of less concern, averaging 2.14 (median 2) and 2.00 (median 2) points, respectively. The Cronbach's alpha coefficient for the questionnaire in this section was 0.803. As depicted in Table 3, significant differences based on gender were observed in the social domain, particularly concerning anxiety levels during the initiation of new friendships, with a greater proportion of females reporting feeling nervous (p=0.0082). Likewise, as shown in Table 4, individuals from areas apart from Northern Thailand expressed increased anxiety regarding this process (p=0.0049). These differences were found to be statistically significant.

Academic

According to Table 2, a significant portion of first-year medical students at Chiang Mai University expressed concern about their upcoming exams, with the highest average rating being 3.66 (median 4). Concerns extended to understanding lecture topics, rated at 3.49 (median 4), and expected academic performance, rated at 3.43 (median 4). Anxiety levels were higher for potential learning compared to teaching participation, with scores of 3.23 (median 3) and 2.65 (median 3), respectively. Some students also felt uneasy about presenting topics or answering questions, mirroring difficulties in understanding lecture content, with scores of 3.14 (median 3) and 3.19 (median 3). Adjusting to the teaching environment and meeting the learning expectations of others was consistently rated at 2.74 (median 3). The Cronbach's alpha coefficient of the questionnaire in this part was 0.937. As depicted in Table 3, male students tended to exhibit less concern across various aspects compared to female students, with statistically significant differences being observed in understanding lecture topics, potential for learning, meeting learning expectations, upcoming exams, presentation of topics, and difficulty of lecture content (p=0.0023, 0.0445, 0.0108, 0.0148, 0.0101, and 0.0111, respectively). Furthermore, according to Table 4, students from non-Northern Thailand showed higher levels of concern across several aspects compared to their northern counterparts, including the upcoming exam and academic results (p=0.0173 and 0.0091, respectively), with these differences being statistically significant.

## Discussion

The examination of survey results from first-year medical students at Chiang Mai University provides valuable insights into the challenges encountered by newcomers as they transition into medical school. Among the four assessed domains - environment, financial, social, and academic - the academic domain emerged as the highest source of concern, while financial concerns received the lowest rating. The data revealed that female medical students reported elevated levels of concern compared to their male counterparts. Additionally, students from Northern Thailand exhibited less concern across nearly all domains in comparison to their peers from other regions. These findings pave the way for a more in-depth exploration of the specific concerns within each domain, shedding light on the distinct challenges faced by first-year students as they adjust to their new academic environment.

Colley outlines the concept of learning transition, emphasizing the following two core dimensions: change and time [9]. These transition dimensions are ongoing, with individuals constantly experiencing various forms of change. These changes can have significant emotional, social, and economic implications, potentially altering both learning trajectories and career paths, as well as influencing the range of choices and opportunities available along the way. Therefore, a transition refers to how individuals respond to changes that take place gradually over time. It occurs when individuals must adapt to new situations or conditions, enabling them to effectively incorporate these changes into their lives [10].

In this study, first-year medical students from the Faculty of Medicine at Chiang Mai University encountered the challenges of higher education, which included multiple concurrent transitions, such as acclimating to a new academic environment, managing an increased workload, and experiencing greater autonomy. These adjustments impacted their transition both positively, by fostering personal growth and new learning opportunities, and negatively, by causing stress and adjustment difficulties. Initially, upon their arrival at the university, students typically felt cheerful and hopeful. However, as they progress through the transition process, they encounter various challenges in the new environment, leading to feelings of depression, confusion, isolation, and increased levels of stress [11]. The findings of this study align with earlier research, illustrating that the stress and anxiety experienced by individuals can be attributed to environmental, financial, social, and academic factors [4]. 

The findings of this study also revealed that the most significant concerns during the transition were the fear of being ignored by their community, followed by adjusting to the new environment and feelings of homesickness, respectively. These challenges tend to arise from the unfamiliar university environment, changes in required learning styles, and the need for time and patience to adapt gradually to the new surroundings during this adjustment process [5,6]. Therefore, educators could provide extracurricular career workshops to offer tailored guidance to students on the university admission process and preparedness, especially for those who come from regions apart from Northern Thailand [5].

The moderate Cronbach's alpha coefficient of 0.655 observed in the environment domain suggests a need for caution when interpreting these findings, as it indicates only acceptable internal consistency. This relatively lower reliability may stem from the multidimensional nature of the construct, which covers diverse aspects such as social separation, homesickness, and adaptation to a new environment. Future research could focus on refining and expanding the questionnaire items to enhance internal consistency and ensure more robust measurement of these experiences. Nonetheless, the significant differences identified across gender and regional groups highlight important variations in students’ transition experiences, offering valuable insights despite this limitation. These findings emphasize the value of tailored support strategies to address the specific needs of different student subgroups during their adjustment to university life.

Although a small number of students faced the financial burden of attending university, the majority expressed greater anxiety about their daily expenses; this study did not identify financial stability or the integrity of medical students as stressors. Implementing an annual grant program for qualified individuals could mitigate financial difficulties [12,13]. This approach would enable the provision of recurring support to individuals grappling with economic challenges. Establishing clear eligibility criteria and transparent distribution mechanisms is critical for guaranteeing fairness and effectiveness [12]. Regular assessment and adaptation of the program may be necessary to meet evolving needs and maximize its impact on the recipients’ financial well-being [12]. However, it is important to note that the low Cronbach's alpha (0.576) for the environment and financial domains indicates limited internal consistency within these measures. The result suggests that the findings in these areas should be interpreted with caution, as the reliability of the data may be compromised. Future research should concentrate on enhancing these measurement instruments to bolster their consistency and validity, thereby facilitating a more precise evaluation of the environmental and financial factors influencing students' stress and well-being.

Assessment and adaptation of the program may be necessary to meet evolving needs and maximize its impact on the financial well-being of the recipients [12]. Participants noted that interpersonal relationships with friends were the most stressful aspect of this transition, followed by seeking advice from teachers and university officials, respectively. Since this was the participants’ first year in medical school, they were still acclimatizing to the university culture, requiring adaptability to navigate the new system and lacking social support [13]. While challenges were evident in forming new friendships and seeking guidance from educators or university staff, other social transition elements, like social isolation and interactions with dorm mates, were not identified as stressors in this study. Therefore, establishing a near-peer support system to offer accessible, informal advice on various aspects of student well-being could facilitate a smoother transition process for students [14].

During the transitional phase of the current academic year, students highlighted the educational aspect as the most stress-inducing factor across various domains. Many students have expressed significant anxiety toward upcoming examinations, understanding lecture materials, and their academic performance expectations. Participants reported difficulties in comprehending lecture material or addressing inquiries, frequently citing the excessive volume of content, unfamiliar pedagogical approaches, or a negative disposition towards the learning process as contributing factors. These difficulties may eventually lead to academic setbacks and failure [15].

Therefore, it is crucial for educators to support students in transitioning from passive knowledge recipients to active and engaged learners [16]. This transition aligns with the principles of adult learning that point out the value of self-regulated learning [17]. Additionally, educators should assist students in setting realistic and attainable learning goals within the medical curriculum framework [18]. This approach would not only promote academic success but also assist in managing self-care and achieving a work-life balance [19].

The findings revealed a correlation between gender and self-perceived concerns, indicating that female students perceived the transition as being more abrupt, necessitating additional time and resilience for adjustment. They initially reported feeling nervous and uncertain, leading to a higher level of perceived stress compared to their male counterparts. The identified subthemes pertained to challenges associated with familial separation, social marginalization, acclimatization to university norms, the establishment of new social connections, comprehension of academic content, expected academic success, and the cultivation of learning potential. These observations align with the previous research conducted by Prowse et al. [20]. Female students frequently exhibit heightened susceptibility to the adverse effects of loneliness on mental health, demonstrating increased levels of depression, anxiety, and stress, especially during the transitional period of the COVID-19 pandemic [19]. Multiple biological mechanisms are believed to contribute to women’s inclination toward depression [21]. These encompass genetically influenced vulnerabilities, fluctuations in hormones linked to diverse aspects of reproductive function, and amplified responsiveness to these hormonal changes within brain systems associated with depressive conditions [21].

Likewise, the results indicated a correlation between geographical regions and self-perceived concerns, suggesting that students from regions apart from Northern Thailand perceived the transition to university life as a major challenge, resulting in a higher level of perceived stress compared to their northern counterparts. The subthemes identified included the challenges of feeling ignored by their community, adapting to university culture, forming new friendships, and comprehending lecture content. These findings are consistent with earlier research by Gray et al. [22]. The primary hurdle encountered by students from regional and remote backgrounds in adapting to university life was homesickness [22]. The highlighted subcategories included acclimatizing to separation from home, integrating into the university culture, and addressing the needs of mature students [22]. Specific challenges encompassed unfamiliarity with campus facilities, adjusting to academic demands, and grappling with the acquisition of new skills [22]. The challenges stemmed from inadequate guidance during the transition period, underscoring the need for educators to implement orientation week initiatives to help students from diverse geographical backgrounds adjust to university life [22]. Likewise, the needs of mature students should be incorporated into tailored, age-appropriate, and family-friendly activities [22].

One limitation of this investigation into the opinions and concerns of students transitioning from high school to medical school is the study design. This is a descriptive cross-sectional study without a qualitative component, which hinders the effective gathering of detailed information and the establishment of causality between the transition process and its predictors. Because there is a month between the start of the semester and the time of data collection, students may not accurately recall their thoughts, feelings, or experiences during that period. They might forget certain details, misremember events, or be influenced by their more recent experiences, leading to inaccuracies in their responses to the self-assessment questionnaire. Such errors could affect the validity of the findings, as the data may not truly represent the students' initial experiences and concerns during the first month of medical school. Additionally, the Cronbach's alpha coefficients for the environment and financial domains were 0.655 and 0.576, respectively, indicating moderate to low internal consistency. This suggests that the items may not consistently reflect the underlying concept of environmental and financial concerns. These limitations warrant caution in interpreting the findings, as they may not fully represent the true nature of the challenges faced by first-year medical students. Future research should focus on refining the survey items to enhance reliability and validity. Despite these limitations, the study achieved a high response rate (60.9%) and displayed high questionnaire reliability (Cronbach’s alpha coefficient 0.935), exceeding 0.90, indicative of excellent reliability. These factors enhance the conclusiveness of the study results. Future research should consider including students in years one and two to provide more comprehensive results.

## Conclusions

The research on the transition of first-year medical students from high school to university underscores the multifaceted challenges they encounter during this critical period of change. As an internal process, the transition involves students adapting to shifts in environmental, financial, social, and academic dynamics, leading to significant emotional and practical adjustments. Challenges, such as adjusting to a new educational system, adapting to cultural norms, forming relationships, facing academic pressures, and managing financial burdens, can lead to stress, anxiety, and emotional responses that negatively impact students’ well-being and academic performance. Collaborative efforts between educational institutions are crucial to supporting students through such transitions, ensuring successful adaptation. Understanding gender and regional disparities in students’ experiences enables the development of tailored support strategies that address individual needs based on gender and geographical backgrounds. Despite the limitations of this research, including its descriptive design and certain exclusions, the high response rate and questionnaire reliability enhance the credibility of the findings. Future research should broaden student inclusion by involving a more diverse and larger group of students, such as students from different years, backgrounds, and regions, to obtain a more comprehensive understanding of their experiences. This approach will help capture a wider range of perspectives and challenges, enabling educators to better support students in navigating the complex transition from high school to medical school, ultimately promoting their academic success and overall well-being.

## Appendices

**Table 5:** The questionnaire for the study on perceptions of the transition process from high school students to first-year medical students. Information for assessing levels of concern on various issues. Scores were rated on a five-point scale, where 5=highest, 4=high, 3=moderate, 2=low, and 1=lowest.

Four aspects of problems	1	2	3	4	5
Domain 1: environment					
(1.1) I feel homesick.					
(1.2) I am afraid of being separated from my family.					
(1.3) I am afraid of being ignored by society around me.					
(1.4) I am afraid that I will not be able to adapt to the university environment.					
Domain 2: financial					
(2.1) I am worried about paying the tuition fees.					
(2.2) I worry about my daily expenses.					
Domain 3: social					
(3.1) I am worried about making new friends.					
(3.2) I am worried about approaching or consulting with a teacher at the university.					
(3.3) I am worried about approaching or consulting university officials.					
(3.4) I am worried about living with dorm mates.					
(3.5) I feel lonely and alone.					
Domain 4: academic					
(4.1) I am worried about whether I will be able to be a part of the teaching or not.					
(4.2) I am worried about adjusting to the teaching environment.					
(4.3) I am worried that I won't be able to understand the content well enough.					
(4.4) I feel like I have to bear the expectations of others when it comes to my studies.					
(4.5) I am worried about my ability to study.					
(4.6) I am afraid the content will be too difficult for me.					
(4.7) I am worried about the upcoming exam.					
(4.8) I am worried about the presentation and answering the teacher's questions.					
(4.9) I am worried about the results not being good enough. Just as expected at the beginning.					
